# Prevalence of self-reported symptoms of sexually transmitted infections, knowledge and sexual behaviour among youth in semi-rural Tanzania in the period of adolescent friendly health services strategy implementation

**DOI:** 10.1186/s12879-018-3138-1

**Published:** 2018-05-19

**Authors:** Ramadhani Abdul, Annette A. M. Gerritsen, Mary Mwangome, Eveline Geubbels

**Affiliations:** 1 Ifakara Health Institute, Ifakara, Tanzania; 2 Epi Result, Pietermaritzburg, South Africa; 3 Ifakara Health Institute, Dar es Salaam, Tanzania

**Keywords:** Sexually transmitted infections, Adolescents, Youth, Health services, Sexual risk-behaviour

## Abstract

**Background:**

Global evidence shows that sexually transmitted infections (STIs) prevalence and sexual risk behaviours are high among youth, and knowledge about STIs is low. In Tanzania, there is limited recent evidence regarding these issues. The aim of this study was to describe the health seeking behaviour of youth reporting STI symptoms in semi-rural Tanzania and to evaluate the association of socio-demographic characteristics, STI knowledge and sexual risk behaviour with STI symptom reporting.

**Methods:**

This was a cross-sectional study involving 2251 sexually experienced youth (15–24 years), who participated in a larger baseline survey of a cohort within Ifakara town. Interview data were electronically collected by trained field workers. Logistic regression analysis was used to identify factors that influence the risk of reporting STI symptoms within the past year, using Stata 12.1.

**Results:**

The prevalence of self-reported STI symptoms in the past year was 19.9%. Almost all of youth had heard of STIs and 32.7% of youth could mention at least one sign. 34.4% had sought care for their STI symptoms, the majority at private facilities. Only 20% of HIV-STI co-infected youth was aware of their HIV status.

Youth with more knowledge of STI symptoms reported to have had symptoms more often (OR = 1.28; 95% CI 1.01–1.62), and those reporting having first sex at 16 or under were more likely to report STI symptoms than those who delayed to 17–19 years (OR 1.27; 95% CI 1.003–1.62).

**Conclusion:**

These findings highlight the need to improve the implementation of Adolescent Friendly Health Services available in Tanzania (especially in semi-rural areas). The inclusion of private facilities and pharmacies in AFHS scale-up would potentially raise the level of STI knowledge, lower the STI prevalence and reduce HIV incidence among youth.

## Background

Studies have shown that Sexually Transmitted Infections (STIs) are a major contributor to acquisition and transmission of HIV [1]. For women, STIs can have additional consequences such as infertility and adverse pregnancy outcomes [2]. In 2004, the WHO estimated that at least 93,000 people died of STIs (excluding HIV) in Africa [3]. Apart from having a health impact, STIs are estimated to account for at least 17% of economic loss due to illness [4] and additional costs due to the cost of treatment [5].

Limited global data are available on the occurrence of STIs among youth aged 15–24 years (UN definition for youth), although some evidence exists that gonorrhoea, but not chlamydia, rates are higher in this age group than in individuals over 24 years [6]. More evidence is available on high risk sexual behaviour among youth, with two reviews, one on Low-and Middle Income Countries (LMIC) [7] and one on sub-Saharan African (SSA) countries [8] showing that high proportions of adolescents (10–19 years) had low condom use, sex before 15 years of age (especially among girls), and multiple sexual partners. With respect to knowledge, most studies focus on HIV, but the few studies from countries in SSA that assessed knowledge of STIs among youth showed an overall low level of knowledge in this population [9, 10].

Data from two large population-based Tanzanian studies done over a decade ago also suggest that this group is at risk of STIs, with the prevalence for Chlamydia, syphilis and gonorrhoea reported to be between 0.4 and 5.0%, and STI prevalence generally being higher among girls than boys [11, 12].

One of these studies [[12] also examined sexual behaviour and found similar high-risk behaviour patterns as seen globally in LMIC / in other SSA countries. Furthermore, fewer than 15% of secondary school students had comprehensive knowledge on STI symptoms [13] and only half of 15–30 year old males and one third of females knew about STI transmission [12].

Over a decade ago Tanzania committed to improving capacity and increasing access of Adolescent Friendly Health Services (AFHS). AFHS represents an approach that brings together all qualities that young people demand to fulfil their health needs [14] and informed Tanzania’s National Adolescent Health and Development Strategy: 2002–2006 [15]. In 2005 Tanzania adopted 7 standards for AFHS, relating to adolescents’ access to information and counselling; access to preventive, promotive, curative and rehabilitative services; adolescents being informed of their rights to AFHS; service providers being able and willing to provide AFHS; supportive policies and systems being in place; health service facilities being organised to provide AFHS and lastly, parents and communities being supportive [16]. However, a recent review showed that some districts did not make substantial progress between 2000 and 2012 with the implementation [17]. The latest Service Access and Readiness Assessment (SARA) reported that the majority of health facilities in Tanzania were offering AFHS, but it also revealed a shortage of staff trained in diagnosis and management of STIs and low numbers trained in AFHS especially in rural facilities [18]. In addition, impact evaluations of the MEMA Kwa Vijana (MkV) intervention - a trial combining education and AFHS conducted from 1999 to 2002 - showed that despite improvements in sexual and reproductive health knowledge, reported attitudes and some reported sexual behaviours, the prevalence of STIs did not decline [12]. Furthermore, the African Youth Alliance (AYA) program, a prevention program for improving adolescent sexual and reproductive health conducted from 2000 among 17–22 year olds found positive impacts on some outcome indicators such as consistent condom use, sexual abstinence in the past 12 months and use of modern contraceptives and this was mostly among females [19].

Age-disaggregated data on STI’s among adolescents are not available from Tanzania’s national health management information system [20]. To our knowledge, no recent results have been reported on STI burden and contributing factors from research studies either. We, therefore, assessed the health seeking behaviour of youth reporting STI symptoms and the association of socio-demographic factors, STI knowledge and sexual risk behaviour with STI symptoms reporting in Ifakara, semi-rural Tanzania, in 2013.

## Methods

### Study design

Data for this cross-sectional study comes from the baseline measurement of the Mzima cohort. This is a prospective cohort including adults aged 15 years and above lodged in the Ifakara Health and Demographic Surveillance System (HDSS), which does repeated serosurveys approximately every 2 years to collect information on HIV, STIs, non-communicable diseases (NCDs), their determinants and related health seeking behaviour. The baseline was conducted from June 2012 to May 2013. Detailed information about the Mzima cohort and Ifakara HDSS profile can be found elsewhere [21, 22].

### Study setting and participants

The Ifakara HDSS is located in Kilombero district in the southern part of Tanzania and the Mzima cohort study population was recruited from the Viwanja Sitini and Mlabani areas, comprising about 5000 households in total. All people aged 15 years and older in the year of the serosurvey were eligible for inclusion (*n* = 9134). A total of 8734 (95.6%) of enrolled participants completed questionnaires. For the current study, all participants aged 15–24 years who ever had sex were selected from the total sample (*n* = 2251; 25.8%).

### Data collection and measurement instrument

To collect the information for the study, a standardised questionnaire translated into Swahili (the language widely spoken in the area) was administered, by means of a face-to-face interview using a tablet personal computer (PC). The methods and tools used in this study were partly adopted from TAZAMA, a project which monitors the health of a population in north-west Tanzania [23]. The interview questions relevant to the current study are those on STI symptoms and knowledge, sexual behaviour and socio-demographic characteristics. First, all participants were asked if they had ever heard about STIs, and for those who responded positively, their knowledge was assessed by asking them to mention any symptoms of STI they knew, without prompting for specific symptoms. After that, the presence of any of the following symptoms (painful urination, urinated blood, genital discharge, genital ulcers or swelling) in the previous twelve months was assessed by active prompting for each of the symptoms, to evaluate the magnitude of self-reported symptoms of STIs. These symptoms were also used by another study [24]. Other information such as access to health services (defined as attending any of the following: inpatient or outpatient hospital visit, visit dispensaries or any health facilities, visited by home-based health care workers), action taken when having STI symptoms, VCT experiences and HIV status was used to characterise the participants who reported STI symptoms.

The two consecutive enzyme-linked immunosorbent assay (ELISA) tests (Vironostika HIV Ag/Ab antigen/antibody and Vironostika HIV Uni- Form II plus 0, ELISA; Vironostika®, Biomérieux BV, Boxtel, The Netherlands) was performed by trained lab technician to screen for HIV. We considered HIV positive samples when the result from the two consecutive test was positive.

### Data management and analysis

All questionnaire data were entered using the Open Data Kit (ODK) technology employed in a tablet PC during data collection in the field. Further data cleaning and data analysis was done using Stata 12 software. The prevalence of self-reported symptoms of STIs was measured as the proportion of those who reported having at least one symptom in the past 12 months out of all respondents. Knowledge of STIs was defined as the ability to correctly state at least one sign of STIs, therefore creating a new binary variable with two values of having and not having knowledge about STI signs. Other STI knowledge variables were “ever heard of condoms” and “ever heard of STI”. The sexual behaviour variables age at first sex, condom use, the age gap between partners, the number of sexual partners in the past year and in their lifetime (one, two, three or more) were included. Note that as participants often had multiple partners, the variable “age gap between partners” was categorized as older by at least five years, younger by at least five years, about the same age and those engaged in sex with partners of different ages (same age, older and/or younger).

The following socio-demographic variables were included: age (15–19, 20–24 years), education level (none/primary level, secondary), marital status (single, married, separated/divorced) and membership of one or more social groups (such as savings group, political party, sports club). Logistic regression was used to assess the association between STI knowledge factors, risk behaviour variables and reporting an STI. An alpha value of 0.05 was used to determine the statistical significance for all analyses. All independent variables with a *p*-value of ≤0.2 at the univariable analysis were added to the logistic regression model, after which other variables were added one by one until the area under the ROC curve did not further improve. Effect measure modification by gender was tested by including interaction terms with the STI knowledge and sexual behaviour variables.

### Ethics

Written informed consent was obtained from all study participants prior to the interview. Data were de-identified to ensure confidentiality of the study participants’ data. The Mzima Cohort was approved by an Institutional Review Board (IRB) of the Ifakara Health Institute (IHI) (Registration number IHI/IRB/No 37–2011) and Medical Research Coordinating Committee (MRCC) of the National Institute for Medical Research of Tanzania (NIMR) (Registration number) (NIMRIl-lQ/R.8aIV0 I. IX/1320).

## Results

### Overall characteristics of the study participants

Table 1 shows the characteristics of the overall study participants and those who reported STI symptoms. The majority of participants were female (68.1%) and the mean age was 20.2 years. Over two-thirds (70.6%) was single. Just less than half of the participants had finished a secondary education or above (48.7%). Only about a fifth (22.3%) were still in school and just over one-third (34.8%) was involved in economic activities that helped the household to generate income. Only 5.5% of youth participated in one or more organised social groups in the area. The population is a mixture of ethnicities, with the Pogoro (22.3%), Ngoni (12.8%) and Ndamba (10.8%) together forming about half of the population.

**Table 1:** Characteristics of the study participants, youth aged 15–24 years (*n* = 2251), Ifakara (Tanzania), 2012/13

Characteristics		All study participants *n* = 2251 (%)	Participants who reported having STI symptoms (*n* = 448)
Socio Demographic			
Gender	Male	718 (31.9)	157 (35.0)
	Female	1533 (68.1)	291 (65.0)
Age [mean (SD)]		Mean 20.2; SD (2.5)	Mean 19.9; SD (2.5)
Marital status	Single	2590 (70.6)	318 (71.0)
	Married	610 (27.1)	121 (27.1)
	Divorced or separated	51 (2.3)	9 (2.0)
Education level	None/Primary	1155 (51.3)	242 (54.0)
	Secondary and above	1096 (48.7)	206 (46.0)
Current activity	In school	482 (21.4)	89 (19.9)
	Work	785 (34.9)	164 (36.5)
	Both	20 (1.0)	7 (1.6)
	None	964 (42.8)	188 (42.0)
Membership of one or more social network	Yes	124 (5.5)	31 (7.0)
	No	2127 (94.5)	417 (93.0)
Ethnicity	Pogoro	501 (22.3)	107 (33.9)
	Ngoni	289 (12.8)	49 (10.9))
	Ndamba	244 (10.8)	43 (9.6)
	Sukuma	169 (7.5)	34 (7.6)
	Hehe	146 (6.5)	28 (6.2)
	Ngindo	141 (6.3)	30 (6.7)
	Mbena	103 (4.6)	31 (7.0)
	Other with frequency < 5%	658 (29.2)	126 (28.1)
Sexual behaviour			
Age at first sex [median (IQR)]		Median 16.0; IQR(15–18)	Median 16; IQR (15–17)
Life time sexual partners			
	One	706 (31.4)	138 (30.8)
	Two	553 (24.6)	100 (22.3)
	Three or more	935 (41.5)	206 (46.0)
	Don’t Know	57 (2.5)	4 (0.9)
Past 12 months sexual partners	None	336 (14.9)	65 (14.5)
	One	1750 (77.7)	340 (75.9)
	Two or more	165 (7.3)	43 (9.6)
Partner age difference(past year)	Partner older by at least 5 years	1036 (54.2)	205 (45.8)
	Partner younger by at least 5 years	152 (8.0)	32 (7.1)
	Partner of about same age	650 (34.0)	128 (28.6)
	Engaged in more than one group	74 (3.9)	16 (4.0)
Condom use last sex with first most recent partner (past year)	Don’t have partner	447 (21.2)	94 (21.0)
	Yes	585 (26.0)	105 (23.5)
	No	1186 (52.8)	248 (55.5)
	Missing	2	1
STI knowledge			
Heard of STI	Yes	1882 (83.6)	388 (86.6)
	No	369 (16.4)	60 (13.9)
STI sign knowledge	Low	1516 (67.4)	284 (63.4)
	High	735 (32.7)	164 (36.6)
Heard of condoms	Yes	2205 (97.9)	438 (97.8)
	No	46 (2.1)	10 (2.2)
HIV status and access to health services			
Ever had VCT	Yes	442 (19.6)	72 (16.1)
	No	1809 (80.4)	376 (83.9)
HIV status (*n* = 2042)	Positive	86 (4.2)	25 (5.9)
	Negative	1956 (95.8)	397 (94.1)
Health service use^a^	Never	714 (31.7)	149 (33.3)
	1–3 visit	1060 (47.1)	212 (47.3)
	4 or more visit	477 (21.2)	87 (19.4)
STI symptoms			
painful urination	Yes	376 (16.7)	376 (83.9)
urinated blood	Yes	84 (3.7)	84 (18.8)
genital discharge	Yes	54 (2.4)	54 (12.1)
genital ulcers or swelling	Yes	89 (4.0)	89 (19.9)

^a^Defined as a number of visit to hospital, dispensary or any health facility in a past year or number visited by home based healthcare workers

**Fig. 1:**
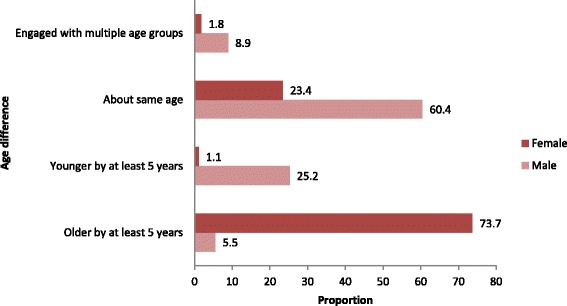
Pattern of Partners’ age difference in a past among youth 15–24 years, Ifakara (Tanzania), 2012/13

With respect to sexual behaviour, the median age at first sex was 16 years with an IQR of 15–18 years. 41.5% reported having had at least three-lifetime partners and 85.0% had had at least one partner in the year prior to the interview. Partner’s age difference differed significantly by gender(chi2 *p*-value< 0.001), with the majority of female youth (73.7%) having had partners who were older by at least five years, whereas male youth engaged more with partners of the same age (60.4%) or those who are younger by at least five years (25.2%) (Fig. 1).

The majority of youth had heard about STIs (83.6%), but only 32.7% was able to mention at least one sign and only 12.2% was able to mention two or more signs. Almost all (97.9%) had heard about condoms.

The prevalence of self-reported symptoms of STIs (having at least one symptom in the past 12 months) was 19.9%. The most common symptom was painful urination (16.7%), others were genital ulcers or swelling (4.0%), genital discharge (2.4%) and urinated blood (excluding menstrual blood for women) (3.7%). 4.7% of youth reported more than one STI symptom.

### Health seeking of participants reported STI symptoms

The majority of youth reported the use of a private pharmacy (79.2%) in the past year for their general health care needs, followed by dispensaries or health facilities (49.1%). Fewer reported using inpatient or outpatient hospital services, 11.8 and 10.3% respectively. About 20% of those who reported STI symptoms were visited by home-based care or community health workers in the year prior to the survey. Some youth reported the use of a traditional healer (13.2%). When asked if they had ever used VCT services in their lifetime, only 16.1% of those who reported STI symptoms ever had VCT services compared to 19.6% in the total group of participants. Only one third (34.4%) of youth reporting STI symptoms in the past year had sought treatment; most of them did so at private clinics or pharmacies (27.3%), followed by government clinics (26.0%). Consulting traditional healers or self-medication for STI symptoms was rare.

In terms of HIV status, the prevalence was higher among this group than among general participants (5.9% versus 4.2%; *p* = 0.01). Of those who reported STI symptoms and were tested HIV positive, very few (20%) were aware of their HIV status.

### Univariable model for factors associated with STI symptoms

Table 2 presents crude and adjusted odds ratios (ORs) for factors associated with STI symptoms.

**Table 2:** Crude and adjusted Odds Ratios (ORs) for factors associated with self-reporting one or more STI symptoms, youth 15–24 years, Ifakara (Tanzania), 2012/13

Characteristics	Unadjusted (Crude) model	Adjusted Model
	OR (95% CI)	OR (95% CI)
Gender		
Male	1	1
Female	0.84 (0.67–1.04)	0.81 (0.63–1.04)
Age		
15–19	1	1
20–24	0.89 (0.72–1.09)	1.15 (0.90–1.47)
Marital status		
Single	1	1
Married	0.99 (0.78–1.25)	1.06 (0.80–1.41)
Divorced or separated	0.86 (0.41–1.78)	0.87 (0.41–1.85)
Education Level		
None/Primary	1	1
Secondary and above	0.87 (0.71–1.07)	0.83 (0.64–1.07)
Current activity		
In school	1	1
Work	1.17 (0.87–1.55)	1.21 (0.85–1.72)
Working and schooling	2.37 (0.92–6.13)	2.27 (0.87–5.95)
Neither work nor school	1.07 (0.81–1.42)	1.17 (0.84–1.62)
Member of one or more social network		
Yes	1	1
No	0.73 (0.48–1.11)	0.76 (0.49–1.17)
Age at first sex		
16 years and under	1.29 (1.03–1.62)*	1.27 (1.003–1.62)*
17–19 (ref)	**1**	**1**
20–24	0.91 (0.52–1.57)	0.92 (0.53–1.51)
Don’t know	0.83 (0.56–1.24)	0.82 (0.55–1.23)
Condom use last sex with first most recent partner		
Yes	1	
No	1.20 (0.93–1.55)	
Don’t have partner	1.12 (0.82–1.52)	
Life time sexual partners		
One	1	
Two	0.91 (0.68–1.21)	
Three or more	1.16 (0.91–1.48)	
Don’t know	0.31 (0.11–0.87)**	
Past 12 months sexual partners		
None	1	1
One	1.01 (0.75–1.35)	0.99 (0.73–1.36)
Two or more	1.47 (0.94–2.28)	1.26 (0.80–1.99)
Partner age difference(past year)		
About same age (reference)	1	
Older by at least 5 years	1.14 (0.85–1.52)	
Younger by at least 5 years	0.98 (0.63–1.54)	
Engaged in more than one group	1.26(0.72–2.22)	
Don’t have a partner	0.96 (0.69–1.35)	
Condom use last sex with first most recent partner (past year)		
Yes	1	1
No	0.75 (0.55–1.01)	0.79 (0.57–1.10)
STI symptoms knowledge		
Low	1	**1**
High	1.25 (1.004–1.54)*	1.28 (1.01–1.62)*
Heard of condoms		
Yes	1	1
No	1.12 (0.55–2.27)	1.27 (0.62–2.68)

Note: * *p* < 0.05; ** *p* < 0.01; Model 1: STI Knowledge, Model2: Risk sexual behaviour and model 3: combined risk sexual behaviour and STI knowledge

Reporting STI symptoms was statistically significant associated with age of first sex (OR 1.29, 95% CI (1.03–1.62) - those who initiated sex at age 16 or below reported more symptoms than those who delayed first sex until 17–19 years of age - and with STI knowledge (OR 1.25, 95% CI (1.004–1.54) - youth who were able to correctly mention at least one sign of STI were more likely to report STI symptoms than those who could not identify one STI sign. All other factors included in the model were not statistically significantly associated with reporting symptoms.

### Multivariable model for factors associated with STI symptoms

In the multivariable logistic model that combined sexual risk behaviour and STI knowledge, STI knowledge continued to be a factor that is independently associated with reported symptoms; youth who were able to correctly mention at least one sign of STIs had a 1.28 higher odds (95% CI 1.01–1.62) of reporting STI symptoms than those who could not mention a single STI sign. Logistic regression also showed that delayed sexual initiation was significantly associated with lower reporting of STI symptoms, in that, youth who initiate sexual activity at the younger age of 16 or under were more likely to report STI symptoms than those delayed to 17–19 years (adjOR 1.27 (1.003–1.62). All other factors were not significantly associated with reporting STI symptoms in youth. Gender did not modify the effect of STI knowledge and sexual behaviour on reported STI symptoms.

## Discussion

The study found an overall prevalence of self-reported STI symptoms of 19.9, and 32.7% of youth had knowledge about STI symptoms. Initiating sex at an early age and having high STI knowledge were both independently associated with reporting STI symptoms among youth.

We found a considerable prevalence of STI symptoms in the past year in the study. However, it is difficult to compare this with other studies conducted in Tanzania or elsewhere, as studies often report on the prevalence of individual STIs [6, 11, 12]. However, the survey conducted in Mwanza region found that 7.6% of males and 11.9% of females had one or more self-reported symptoms that could be due to an STI [11]. However, those percentages related to symptoms at the time of interview whereas our study asked about symptoms in the past year, which might explain why we found a higher prevalence.

Our study has found that knowledge about STI symptoms in the youth population was very low, despite the fact that a large number had heard about STIs. The study conducted among secondary school students in Dar es Salaam found that 78.6% of boys and 82% of girls knew at least one symptom [13]. The higher proportion may be because of study setting (urban) and population (students only) leading to better access to information. Our finding that there was low STI symptom knowledge among youth was, however, consistent with multiple other studies [9, 10, 12], but differences in the magnitude of STI knowledge may be due to the definition and criteria used to define knowledge by different authors in different studies. For example, a study of youth and young adults aged 15–30 years conducted in rural Tanzania [12] defined STI knowledge as the ability to correctly respond to three questions concerning STI acquisition.

Those who start engaging in sex at a young age are also at higher risk of getting STIs. The link between the age of sex initiation and subsequent risk of STIs has been highlighted in a number of previous studies [25, 26]. One study found that youth who initiate sex at an early age were more likely to engage in high-risk sexual practices that increase the chance of contracting an STI [25]. In addition youth, especially girls, are generally considered to have lower power for negotiating safe sex (such as condom use) which makes them more vulnerable to STIs [13].

When assessing the association between STI knowledge and risk of reporting STI symptoms, our findings showed that youth who had higher knowledge were more likely to report STI symptoms. A study conducted in Nigeria also found that a higher proportion of respondents with good knowledge of STIs reported symptoms [27]. In a setting such as ours where specific sexual health education is difficult to access, it is possible that youth who have experienced symptoms of STIs before have gained knowledge about these afterwards; hence there would not be a protective effect of STI knowledge on reporting symptoms.

Our findings may be puzzling given the fact that numerous efforts (such as AFHS, community and school sexual health education programs) were established nationally in order to improve knowledge of and access to sexual health promotion and services [12, 15, 19]. Chandra-Mouli and colleagues concluded that efforts at the national level may not yet have translated into practice [17] – indeed there has been limited implementation of AFHS in the two hospitals in the Ifakara area (Personal communication District health official). Research elsewhere in Tanzania has shown that reasons for poor implementation include the unavailability of staff, buildings, guidelines not being well publicised, lack of confidentiality, service providers not having access to the documents, condom shortage [28, 29] and action should be taken on this in order to improve service provision.

Furthermore, for the most part, sexual and reproductive health education is left to staff who work at the HIV care and treatment clinic (CTC) or within the hospitals, although some community education work has been done by an international NGO in collaboration with two district departments. Our finding that two-thirds of youth with STI symptoms had not sought care underscores the continued need for community education to promote care seeking for STIs.

An additional explanation may be that services may be available where youth do not seek them. The majority of youth and adolescents in our study preferred going to private facilities when seeking care for health problems, including STI symptoms rather than to government health facilities. While this study did not ask for the reasons, other studies have listed easy access within the community, and confidentiality and trust of pharmacists as among the reasons [30, 31] despite acknowledging that the majority of pharmacists had little knowledge of STIs and their syndromic management [30, 32]. This finding has significant implications for adolescent health policy in Tanzania. It suggests that to reach youth, health planning and programs must increase the involvement of private pharmacies and clinics. This would require adaptation of the pharmacy pre-service training curriculum to include youth friendly training components especially on risk assessment, referral needs and syndromic management for STIs, as well as regulation and quality control of community pharmacies. With respect to HIV testing services, only about 20% of HIV infected youth who reported STI symptoms were aware of their HIV status, and the prevalence of HIV was significantly higher among this group than among general participants (5.9% versus 4.2%; *p* = 0.01). The association between STI and HIV acquisition has widely been documented [33, 34] and the presence of STIs is linked with transmission and progression of HIV [34]. The finding that the majority of HIV infected youth were unaware of their HIV status would mean that they are an important reservoir for transmission, because they are more infectious because of their STI positive status, compared to STI negative-HIV positive youth.

The finding shows that youth are at increased risk for HIV as a result of untreated STI and therefore emphasis must be put on creating youth-friendly case management of symptomatic STI at the community and health facility level in order to reduce the incidence of HIV in this age group.

This study has some limitations. Firstly, self-reported STI symptoms were used as a proxy for STI prevalence and not the laboratory diagnosis. This might have led to an underestimation of the STI prevalence, as cases, especially women, might be asymptomatic or youth do not dare to indicate they have these symptoms. On the other hand, the non-specificity of some of the symptoms may have led to an overestimation of STI prevalence. In a study comparing the syndromic versus laboratory diagnosis of STIs among women in the North of Tanzania, the specificity of the symptoms we asked for ranged from 94 to 99%, whereas their sensitivity ranged from only 2 to 7%, making it much more likely that our findings are an underrepresentation rather than an overestimation of the true burden of STIs in our study population [24]. Secondly, only awareness of STI symptoms was used to define knowledge, but youth might have knowledge about prevention of STIs, transmission, risk factors, or treatment and thirdly, this study did not collect information on STI experiences among youth; self report of STI symptoms may have been influenced by past experience of STI.

## Conclusion

In conclusion, youth frequently reported STI symptoms and high risk sexual behaviour, yet had very low knowledge of STIs. Youth frequently seek care for health problems, including for STIs, outside of government health facilities.

These findings highlight the need to improve the implementation and reach of Adolescent Friendly Health Services available in Ifakara specifically, but possibly also in the country in general. Stronger youth education on STI symptoms and inclusion of private facilities in AFHS might be needed in order to raise the level of STI knowledge, lower the STI prevalence and reduce HIV incidence among youth.

### Key message

- There is a high reported prevalence of STI symptoms in youth in semi-rural Tanzania.
- The knowledge of STI among youth is low despite risky sexual behaviour
- The prevalence of STI symptoms was linked to the early age of sexual intercourse and STI knowledge.
- The majority of youth and adolescents preferred going to private facilities or pharmacies when seeking care for health problems including STI.

## Abbreviations

AdjOR: Adjusted odds ratio
AFHS: Adolescent friendly health service
AYA: African Youth Alliance
CTC: Care and treatment clinic
ELISA: Enzyme-linked immunosorbent assay
HDSS: Health and demographic surveillance system
HIV: Human immunodeficiency virus
IHI: Ifakara Health Institute
MkV: Mema Kwa Vijana Trial
NCDs: Non- communicable disease
NIMR: National Institute for Medical Research
ODK: Open data kit
OR: Odds ratio
PC: Personal computer
ROC: Receiver operating curve
STI: Sexually transmitted infection
UN: United Nations
VCT: Voluntary counselling and testing
WHO: World Health Organisation
